# Systematic review of sensory-based interventions for children and youth (2015–2024)

**DOI:** 10.3389/fped.2025.1720179

**Published:** 2025-11-13

**Authors:** Aimee Piller, Jessica McHugh Conlin, Tara J. Glennon, Lauren Andelin, Kelly Auld-Wright, Krysti Teng, Talicia Tarver

**Affiliations:** 1 Piller Child Development, LLC, Phoenix, AZ, United States; 2 Healthy 360, Sergant Bluff, IA, United States; 3 Occupational Therapy, Quinnipiac University, Hamden, CT, United States; 4 Occupational Therapy, Virginia Commonwealth University, Richmond, VA, United States; 5 Occupational Therapy, Keck Graduate Institute, Claremont, CA, United States; 6 Health Science Library, UCLA Lab School, Los Angeles, CA, United States

**Keywords:** sensory, systematic review, sensory-based intervention, sensory processing, developmental disorder

## Abstract

**Introduction:**

Children with developmental disorders often benefit from interventions supporting participation, including sensory interventions, which should be grounded and informed by evidence. The purpose of this systematic review was to evaluate and summarize current evidence on the effectiveness of sensory-based interventions (SBIs) on functional outcomes to guide interventions.

**Methods:**

Searches were performed in Medline (OVID), CINAHL Complete, PsycINFO, OTSeeker, Cochrane Reviews, and ERIC. Inclusion criteria included the following: published in English between May 2015 and January 2024; participants aged 0–21 years with sensory integration/processing challenges; level I and II studies as classified by Johns Hopkins Nursing Evidence-Based Practice Model; functional outcomes indicated. The review followed the guidelines of Preferred Reporting Items for Systematic Reviews and Meta-Analyses.

**Results:**

Twenty-one studies were included. Strong strength of evidence supported use of deep pressure tactile input and caregiver training on the use of sensory strategies. Moderate strength of evidence supported that alternative seating did not improve attention. Additionally moderate strength of evidence supported targeting a variety of sensory systems is more effective than targeting only one system. There is a lack of evidence on the impact of sensory environmental modifications.

**Discussion:**

SBIs may be useful for improving functional outcomes and participation. Further research is needed to clarify effectiveness for specific outcomes.

## Introduction

Sensory integration and processing (SI/SP) differences are estimated to affect 5%–25% of children in the United States (1, 2). The prevalence is higher in clinical populations such as children diagnosed with autism spectrum disorder (ASD) (3), children with attention deficit hyperactivity disorder (ADHD) (4), and other developmental disorders such as fetal alcohol spectrum disorder (5), and Down syndrome (6). SI/SP differences vary and may or may not impact participation in functional activities (7). When these differences do impact participation, they may be delineated from SI/SP differences and referred to as SI/SP challenges, which is the terminology used in this manuscript. Sensory-based interventions (SBIs) are commonly used to support participation in daily activities for individuals with SI/SP challenges. Occupational therapists are widely recognized as leaders in the evaluation and treatment of SI/SP challenges (8, 9). Therefore, children with SI/SP challenges are frequently referred to occupational therapy when these challenges interfere with daily participation. Through comprehensive evaluations, occupational therapists design interventions that promote meaningful participation in everyday (10).

The aim of SBIs is to temporarily modify a child's physiological arousal level, creating a better match between the person and task, with the goal to improve behavior and participation in tasks (11). SBIs are informed by sensory integration theory, but they have clearly delineated differences from direct, one-on-one occupational therapy intervention using Ayres Sensory Integration® intervention, termed ASI (10, 11). Key features of ASI include advanced therapist training and mentorship and adherence to fidelity principles, including active engagement of the child, individually tailored activities, and play opportunities presented at the just-right challenge to facilitate adaptive responses (12, 13). In contrast, SBIs tend to be practitioner or adult-led interventions, involving passive sensory input applied to the child, and can include sensory techniques, such as massage, wearing earmuffs, passive swinging, caregiver sensory training, and sensory environmental modifications (10, 11). SBIs may be implemented by caregivers and other team members or accessed independently by the child as part of an occupational therapy intervention plan in natural environments such as the home, community, and schools.

Two systematic reviews published in 2015 examined the use of SBIs with autistic populations. Watling and Hauer (14) reviewed 23 studies and found mixed evidence for the effectiveness of SBIs, including limited evidence on the effectiveness of sound therapies and no significant effect with the use of alternative seating. Overall, weighted vest studies demonstrated some improvements with attention, but overall evidence of their effectiveness was insubstantial. Linear vestibular movement demonstrated improvement in responding to questions, though the sample size was small. This review also included one study of the sensory environment showing positive effects of sound absorption and lighting on attending behaviors. Case-Smith and colleagues (11) conducted a systematic review including 14 studies between 2001 and 2011 examining SBIs. Interventions in this review included weighted vests, brushing protocols, sitting on therapy balls, and other multi-sensory strategies; however, the rigor of the studies was compromised by the lack of specified protocols and/or manualized descriptions of the intervention and control conditions, resulting in mixed results as to effectiveness.

Two additional systematic reviews examined the evidence of the use of SBIs for children with SI/SP challenges beyond autistic populations. Bodison and Parham (15) included studies using sensory techniques and sensory environmental modifications, although not all outcomes were participation-based. The strongest evidence was found for Qigong sensory therapy for symptom reduction, decreasing parenting stress, and language skills. However, limited evidence was found for determining the effectiveness of weighted vests on in-seat behaviors and attention. This review did not find any significant positive effects of linear swinging for tabletop task behaviors, nor did it identify better play skills in the treatment group vs. the control for sensory-rich classrooms. Further, the sensory environmental modifications reviewed included only one study, which demonstrated significant improvements in participation within a dental environment. However, in this dental environment study, deep pressure touch was also provided to the participants in addition to the environmental modifications, making it difficult to determine which intervention had the greatest effect.

The second systematic review intended to examine the evidence of parent and teacher education and coaching for children with SI/SP challenges beyond the autistic population (16). This purpose of this investigation was to analyze evidence of the efficacy of parent or teacher training/coaching., the studies that met the inclusion criteria only included parents of children who all had a diagnosis of autism and none included teachers. The review included four studies that demonstrated improvements in parental stress or child performance/behaviors, but did not include evidence of SBI effectiveness.

Sensory integration and processing practice guidelines for children and youth with SI/SP challenges, published by AOTA (17), reported strong to moderate strength of evidence for the following interventions: Qigong massage to improve self-regulatory behaviors, sensory-adapted dentist office to reduce distress and discomfort for children with ASD, parent coaching to reduce stress and improve child behaviors, Alert Program® for self-regulation to improve executive function for children with fetal alcohol syndrome, and horseback riding to improve social functioning for children with ASD.

Since the release of the 2018 AOTA practice guidelines (17) and systematic reviews on SBIs, health and education fields have increasingly emphasized holistic, neuro-affirming approaches, moving away from interventions aimed primarily at symptom reduction. Further, interventions for individuals with developmental disabilities have increasingly moved away from deficit remediation toward a strengths-based approach that emphasizes identifying and building upon existing abilities. This approach functions under the assumption that people are unique and competent, have values and preferences, and build their lives around strengths rather than weaknesses (18). As part of the shift to more neuro-affirming intervention approaches, it is important to promote autonomy and independence, directed towards building strength rather than reducing symptoms. Therefore, this systematic review considered outcomes related to functional performance and participation and excluded studies that focused only on the outcome of symptom reduction.

The aim of this systematic review was to identify and examine literature on SBIs published between June 2015 and January 2024 and to answer the following question:

What is the effectiveness of specific sensory-based interventions (including sensory techniques, caregiver-focused sensory interventions, and sensory environmental modifications) to support functioning and participation for children and youth (0–21 years old) with SI/SP challenges that interfere with everyday life participation?

For this review, we defined SBIs as three distinct interventions as follows (17):

- Sensory technique: application of sensory stimuli through materials, tools, and activities applied directly to the child or through positioning a child on or in a device (e.g., weighted items, passive swinging) or accessed directly by the child.
- Caregiver-focused interventions: working with a child's caregiver (anyone who attends to or looks after a child including parent, guardian, teacher, etc.) to provide support for the sensory needs of a child who exhibits SI/SP challenges within the natural context to support participation of the child and group of which the child is a member.
- Sensory environmental modification: change in the intensity, complexity, or quality of one or more sensory elements in the ambient physical environment surrounding the child to support participation.

## Methods

The systematic review followed the Preferred Reporting Items for Systematic Review and Meta-Analysis (PRISMA) guidelines (19). Prior to initiation, the review protocol was reviewed by an independent methodologist.

### Information sources and search strategy

The search strategies were developed by a team of experts in SI/SP together with the Health Science Librarian at Virginia Commonwealth University and built upon the search terms and strategies from the most recent systematic reviews for SBI (11, 14–16). Search terms included sensory processing, sensation disorder, sensory integration disorder, and related terms (see Table 1 file for full search terms). The review included six databases chosen based on past reviews and expert opinion of librarian: Medline (OVID), CINAHL Complete, PsycINFO, OTSeeker, Cochrane Reviews (trials only), and ERIC. Search terms were based on the research questions and identified using a Medical Subject Headings (MeSH) term search. The MeSH and keyword search strategy was translated for each of the other databases with their respective controlled vocabularies.

**Table 1:** Search terms.

Question	Search terms
Population/Problem	Sensation disorders, somatosensory disorders, sensory integration disorders, sensory processing disorders, sensation disorders, somatosensory disorders, clumsy child syndrome, developmental coordination disorder, developmental dyspraxia, disorder of attention, motor, and perception, fine motor deficits, gross motor deficits, learning disabilities, nonverbal learning disorder, perceptual motor deficits, regulatory disorder, sensory integrative dysfunction, sensory modulation disorder, sensory modulation dysfunction, sensory motor deficit
Intervention	Activities of daily living, activity, activity groups, adaptive behavior, adaptive equipment, assistive technology, Astronaut training, attention, auditory integration training, augmentative communication, ball chairs, bilateral coordination, bilateral intervention, coaching, cognitive-behavioral therapy, cognitive intervention, consultation, context, contextual, CO-OP, decision-making skills training, early intervening, early intervention, emotional regulation, employment, environment, environmental modification, executive function, exercise, family centered care, family coping, coping skills, family interaction/participation, friendship, friendship group, functional approaches, handwriting, instrumental activities of daily living, integrated listening systems, job coaching, job training, Kawar protocol, leisure, life coaching, massage, motor planning, multisensory integration, natural environment intervention, neurodevelopmental treatment, neuromotor occupational therapy, occupational therapy, occupation-based, ocular motor skills, oral sensorimotor programs, parent/teacher mediated, parent training, peer group, peer interaction, peer mediated, perceptual motor learning, play, praxis, pressure vest, prevocational, priming, problem-solving skills training, relationship-based intervention, rest, routines-based interventions, self-care, self-management, sensory diet, sensorimotor integration, sensory integration, sensory integrative, SI, sleep, social competence, social participation, social skills training, social stories, strengths-based, supported education, supported employment, tactile stimulation, therapeutic listening, time management, touch pressure, transitioning, transitions, vestibular stimulation, weighted blankets, weighted items, weighted materials, weighted vests, Wilbarger protocol, work, yoga
Study Design	Appraisal, best practices, case control, case report, case series, clinical guidelines, clinical trial, cohort, comparative study, consensus development conferences, controlled clinical trial, critique, crossover, cross-sectional, double-blind, epidemiology, evaluation study, evidence-based, evidence synthesis, feasibility study, follow-up, health technology assessment, intervention, longitudinal, main outcome measure, meta-analysis, multicenter study, observational study, outcome and process assessment, pilot, practice guidelines, prospective, random allocation, randomized controlled trials, retrospective, sampling, scientific integrity review, single subject design, standard of care, systematic literature review, systematic review, treatment outcome, validation study, nonconcurrent, multiple baseline, experimental study, RCT, explanatory mixed methods, quasi-experimental, nonexperimental, exploratory, convergent, multiphase mixed methods, qualitative studies

Studies that included sensory integration but with no mention of fidelity to ASI were included as a sensory technique.

### Inclusion/exclusion criteria

Studies were selected based on the following inclusion criteria: (1) peer-reviewed articles published between May 2015 and January 2024; (2) participants included children (ages birth to 21 years) with documented SI/SP challenges identified by psychometrically sound assessments, including disorders that are commonly associated with SI/SP challenges such as ASD, ADHD, and developmental coordination disorder (DCD) with documented SI/SP challenges; (3) published in English; and (4) have participation or occupation-based outcomes. This review included Hopkins Level I, II, and III studies, according to Johns Hopkins Nursing Evidence-Based Practice Model (20). Single-subject design (SSD) studies were considered as Level II as long as they met the criteria of Logan Level I–IV (21) classification for SSD studies (20). Inclusion criteria for SSD also required having at least three participants and at least two treatments compared to a baseline or at least three treatments compared to each other. Studies were excluded if outcomes were unrelated participation, such symptom reduction. Interventions that included terms such as “sensory integration” or “sensory integration therapy/treatment” with no mention of the ASI Fidelity Measure (13) were considered SBIs rather than direct, one-one one ASI intervention and, therefore, were included within this SBI study.

### Study selection

The research team consisted of occupational therapists with expertise in SI/SP. Articles meeting the search terms were imported into Covidence, an online software to manage systematic reviews and allow reviewers to screen articles at various phases while remaining blinded to one another. Exact duplicates were removed by the Covidence system, and reviewers removed any additional duplicates. Initially, the titles were screened to eliminate any articles that were irrelevant to the search question and overall project (out of date range, not research articles, not related to sensory interventions, etc.). After the initial title screen, abstracts were independently reviewed by two researchers, with a third serving to resolve any conflicts. Based on the abstract review, any clearly non-relevant articles were eliminated based on the inclusion/exclusion criteria. The librarian then obtained the full-text articles, which were independently reviewed by two researchers, with a third researcher serving to resolve any conflicts. All decisions, including reasons for exclusion, were noted in the master citation table (see Supplementary File).

### Extraction and synthesis

Extraction included levels of evidence, research design, population, inclusion criteria, dose, intervention, outcomes, and results (see Supplemental File) for each of the studies. Two researchers completed the extraction, with a third reviewing for accuracy.

### Analysis

The levels of evidence were determined by using the Johns Hopkins Nursing Evidence-Based Practice Model (20) and the strength of evidence was evaluated utilizing the AOTA (22) *Guidelines for Systematic Reviews* (see Table 2). Strong evidence was identified as having at least two studies identified as a Hopkins Level I study. Moderate evidence was identified by having at least one Hopkins Level I and multiple Hopkins Level II, which included Logan Level I–IV. Low evidence was classified by studies below Hopkins Level II or Logan Level IV and studies with significant flaws or high risk of bias. Risk of bias was assessed by two reviewers, blinded to one another, using the Cochrane tool for controlled trials (23) and the National Heart Lung and Blood Institute (24) for studies without a control group.

**Table 2:** Strength of evidence.

Level	Description
Strong	Two or more Level I studies The available evidence includes consistent results from well-designed, well-conducted studies. The findings are strong and unlikely to be called into question in future studies
Moderate	At least one Level I study or multiple high-quality Level II The available evidence is sufficient to determine the effects on health outcomes, but confidence in the estimate is constrained by factors such as: - The number, size, and quality of individual studies - Inconsistency of findings across the individual studies As more information (other research findings) becomes available, the magnitude or direction of the observed effect could change, and this change may be large enough to alter the conclusion related to the usefulness of the intervention
Low	Small number of low-level studies, flaws in the studies, and so on The available evidence is insufficient to assess effects on health and other outcomes of relevance to occupational therapy. Evidence is insufficient because of - a limited number or size of studies, - important flaws in study design or methods, - inconsistency of findings across individual studies, and - lack of information on important health outcomes. More information may allow estimation of effects on health and other outcomes of relevance to occupational therapy

AOTA (22).

## Results

**Figure 1:**
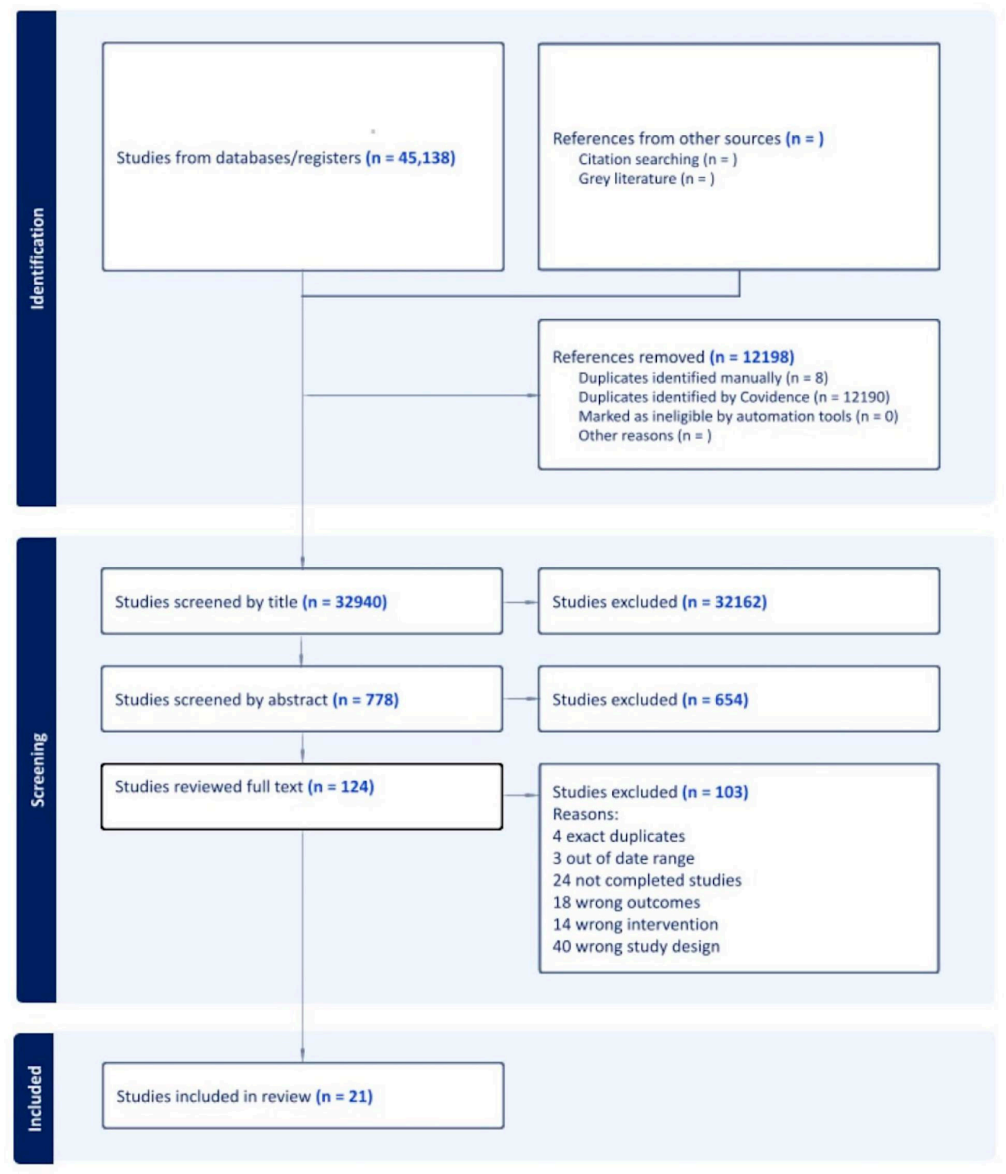
PRISMA flow chart.

The initial search resulted in 45,138 articles with 12,198 initially identified as duplicates. After duplicates were removed, 32,940 article titles were screened for relevance and inclusion criteria. Of the original set, 32,162 were excluded based on the title, leaving 778 articles. These 778 abstracts were independently reviewed by two researchers. The two parties agreed upon 582 articles, with the remaining 196 requiring a review by a third researcher to reach agreement. In total, 654 were excluded at the abstract level, leaving 124 full-text articles. Of the 124 full-text reviews, 103 were excluded. The PRISMA Flowchart outlining the selection process, including reasons for exclusion, can be found in Figure 1. A total of 21 articles met the inclusion criteria and were, therefore, included in this review. An evidence table outlining all the details of the studies included in this review are included in a Supplementary File.

### Study characteristics

Studies were initially organized by setting and population. Settings included school (*n* = 7), therapy clinics (*n* = 5), participant's home (*n* = 3), clinic and home (*n* = 4), home and school (*n* = 1), and dental clinic (*n* = 1). Six hundred seventy-five participants were represented in the studies. The populations represented in the studies included children with various diagnoses, including ASD (*n* = 7 studies), ADHD (*n* = 7 studies), developmental delay (*n* = 2 studies), and sensory processing disorder (SPD) or sensory processing concerns (*n* = 8 studies). Participant ages ranged from one year to 21 years of age. Of the 21 studies, eight took place in the United States, three in Canada, three in India, and one study took place in each of the following countries: Iran, Turkey, Sweden, Taiwan, Finland, Israel, and Japan. Various outcome measures were utilized in the studies and included outcomes related to attention, task completion/productivity, quality of life, motor skills, occupational performance, sleep, Frankl scale, knowledge of sensorimotor strategies, behavior and emotional functioning, and goal attainment. Of the 21 studies, five were Hopkins Level I studies and 16 were Hopkins Level II studies. Of the 16 Level II studies, six were single-subject designs of Logan Level IV or higher.

### Sensory-based interventions

Three main concepts were articulated in the articles, each addressing one aspect of SBIs outlined in the review question: sensory techniques, caregiver-focused interventions, and sensory environmental modifications. Four main sensory techniques were identified in the studies: deep pressure tactile input, alternative seating, sensory input targeting multiple sensory systems (more than two sensory systems), and sensory interventions targeting one or two sensory systems. Caregiver-focused interventions were identified as caregiver education and home-based strategies. Only one study that addressed sensory environmental modifications met the inclusion criteria.

There is strong strength of evidence for deep pressure tactile input positively impacting functional outcomes (25–28). Four studies supported the use of deep pressure tactile stimulation, two of which examined weighted items and two of which examined massage. A Level I study (26) with moderate risk of bias demonstrated statistically significant improvements in motor skills for young children who received massage in addition to routine rehabilitation. Outcome measures for this study included *Comprehensive Development Inventory for Infants and Toddlers-Diagnostic Test* (29), to assess motor, language, self-help, and social skills, and a non-standardized sleep questionnaire. Weighted vest wearing did not significantly improve attention, body perception, coping skills or learning when worn in the school setting, as demonstrated by a Level II study with low risk of bias (27). This study did not indicate activities in which participants engaged in when wearing the weighted vest. Therefore, if participants were moving while wearing the weighted vest, this intervention may have also included proprioceptive and vestibular input in addition to deep pressure tactile input. The outcome measures included surveys of teachers and parents to assess the child's attention, body perception, coping skills, and learning. Two studies focused on the outcome of sleep with both showing statistically significant improvements in sleep (25, 28). The first Level II study (28) with a low risk of bias utilized massage with joint compressions prior to bedtime. Participants had significant improvements in overall sleep and a decrease in daytime sleepiness. Outcome measures for this study included Goal Attainment Scaling (*GAS*) (30) and the *Child Sleep Habits Questionnaire (*31) to assess sleep*.* The second study utilized a weighted blanket for sleep (25). This Level I study with a low risk of bias yielded statistically significant results for improved sleep for children with ADHD. This study used an actigraph and a sleep questionnaire as the outcome measures used to assess sleep quality.

There is moderate strength of evidence that alternative seating does not show significant improvement in functional behavior (32–34). Three studies addressed the use of alternative seating to improve attention. All three studies were Level II with two being SSD. One study (34) examined the use of stability balls in the school setting and found no significant improvements in productivity or seatwork. In another study in the school setting (32), participants did not show any significant improvements in attention while sitting on cube chairs or T-stools. The third study (33) examined the use of stability balls in the home setting and found no statistically significant improvements on attention or behavior. The risk of bias was moderate for all studies. All studies created their own operational definitions for observable behaviors and used frequency counts as the outcome measure to assess attention and behavior.

There is moderate strength of evidence that sensory techniques targeting an increased input to multiple sensory systems can impact functional behaviors (35–39). In all studies, various types of sensory input targeting multiple sensory systems (i.e., vestibular, proprioception, tactile, visual, etc.) were utilized with the goal of increasing functional outcomes. The five studies addressed the use of sensory techniques for functional outcomes. Three Level II SSD studies examined the use of multiple sensory techniques within the regular routine of the classroom, each with moderate risk of bias. For the first study (35), one of three participants showed significant improvements for in-seat behaviors. The second (36) showed significant improvement in task completion for four of seven participants. A third Level II (38) SSD study using sensory diets as part of the regular school day showed three of four participants improving behavior. While results were varied, each participant showed significant improvement in at least one phase of the intervention. All the studies used frequency counts of targeted observable behaviors as their primary outcome measure.

In settings other than schools, a nature-based sensory program used in conjunction with sensory interventions (39) found statistically significant improvements in functional performance as compared to the control group that only received sensory interventions. This was a Level II study with a moderate risk of bias. The *Weiss Functional Impairment Rating Scale* (40) served as the primary outcome measure for functional performance. In a Level II study with high risk of bias (37), sensory interventions, when used with behavioral interventions, were shown to have a greater effect on functional performance in toileting as compared to behavioral interventions alone. This study used frequency counts of toileting and the *Canadian Occupational Performance Measure* (*COPM)* (41) as primary outcome measures to assess participation in toileting.

There is moderate strength of evidence from one level I and two level II studies (42–44) for SBIs aimed at only one or two sensory systems. The Level I RCT study (42) with low risk of bias using movement on a treadmill with vibration showed statistically significant results for quality of life, attention, and executive functioning. However, this study did not show any differences in these areas between individuals who used the treadmill only and those who used the treadmill with vibration. Outcome measures included the *Pediatric Quality of Life Inventory* (*PedsQL)* (45), to assess quality of life, *Behavior Rating Inventory of Executive Function* (*BRIEF)* (46) to assess executive functioning, and the *Conner's Rating Scale* (47) and *Stroop test TBAG form* (*STP-TBAG)* (48) to assess attention. A Level II SSD (43) with moderate risk of bias study examining the use of fidget spinners indicated that participants who used fidget spinners had no significant improvements in gross motor movements (as measured by accelerometers worn by participants) and instead demonstrated increased violations of attention as compared to the control. Frequency counts were the primary outcome measure used to evaluate the effectiveness of attention. Finally, a Level II crossover experimental design study (44) with low risk of bias showed significant improvements in functional performance related to auditory stimuli for autistic children using earmuffs, whereas the use of noise-canceling headphones did not demonstrate significant results. This study used *GAS* (30) as the primary outcome measure to assess functional behaviors related to auditory stimuli.

There is strong strength of evidence to support sensory input provided through caregiver education and home-based strategies as effective in improving functional performance and participation in daily activities (49–53). There were five studies that addressed caregiver education and using home-based sensory strategies after parent training to improve occupational performance. A Level II study (49) with a moderate risk of bias revealed statistically significant improvements in occupational performance as measured by the *COPM* (41) for children with sensory concerns. Two Level II studies, one with a low risk of bias (50) and one with a moderate risk of bias (51), showed a significant increase in knowledge of sensory strategies but no significant or negative impact on functional outcomes. Both studies used the *Sensory and Motor Strategies Questionnaire* (50) as an outcome measure. The study by Mah and Doherty (50) also used the *COPM* to assess occupational performance, while the study by Mah and colleagues (51) included frequency counts as an additional outcome measure for visual attention. A Level I study (52) with moderate risk of bias found that standard therapy (speech and language and behavioral therapy), when coupled with implementation of home-based sensory interventions resulting from caregiver training, resulted in statistically significant improvements in quality of life and behavioral and emotional functioning. The study used the *PedsQL* and *Children's Global Assessment Scale* (54) as outcome measures to assess quality of life and behavior and emotional function. Finally, another Level I study (53) with a moderate risk of bias indicated a program for children with ASD that focused on caregiver sensory knowledge, coaching, and support had statistically significant improvements in functional performance. This study used the *COPM, GAS,* and *Parenting Sense of Efficacy Measure* (55) as outcome measures to assess occupational performance and parent efficacy.

There is not enough evidence to conclude the effectiveness of sensory environmental modifications. Only one study (56) explored sensory environmental modifications. This Level II study had a moderate risk of bias and examined the impact of sensory environmental modifications to a dental office on patient participation within the dental office setting. Significant differences in positive behaviors were found between the patients in the modified sensory environment and the regular dental environment as measured by the *Frankl Scale* (57) to assess behaviors in a dental office.

## Discussion

This systematic review included a variety of SBIs, which were grouped into three categories: (1) *sensory techniques*; (2) *caregiver-focused interventions*; and (3) *sensory environmental modifications*. Consistent with the findings of previous systematic reviews (11, 14–16), the current review also found mixed evidence regarding the effectiveness of SBIs. Specifically, the only *sensory technique* with strong evidence is the use of deep pressure tactile input. There is also strong strength of evidence for *caregiver training* on the use of sensory techniques. There is moderate evidence that using a variety of sensory techniques to target multiple sensory systems is more effective than targeting only one system at a time. However, this is also dependent on the reason for implementing the sensory technique. For example, interventions targeting the auditory system were more effective for activities involving auditory input than using a tactile-based strategy, such as a fidget, to support attention. Finally, there is not enough research to make any determinations on the effectiveness of sensory environmental modifications.

Sensory techniques in this review encompassed a variety of interventions targeting various sensory systems, including vestibular (42), auditory (44), tactile (26, 43), and interventions designed to target multiple sensory systems, including vestibular, proprioceptive, and tactile (35, 36, 38). Descriptions of the interventions were not always detailed, especially when more than one sensory system was targeted. The lack of uniformity in the implementation of SBIs and the lack of clearly defined goals and outcomes for the use of SBIs resulted in varied results as to the effectiveness of sensory techniques. It is also worth noting that many of the outcome measures included more subjective forms of assessment, such as GAS, which are not without bias (58, 59) and may be more subjective in nature. In addition, many of the studies had small sample sizes, which limited their generalizability.

Several studies used multiple sensory interventions targeting different sensory systems, making it difficult to isolate which SBI was responsible for the observed changes. While it is typical in clinical practice to implement more than one intervention or more than one SBI, it limits the ability to document precise research outcomes. Notably, Ikuta and colleagues (44) and Lönn and colleagues (25) presented stronger evidence of effectiveness was indicated when sensory techniques targeted specific sensory systems related to participation goals, and in studies that incorporated more than one targeted sensory system, such as in Benson and colleagues (35) and Pingale and colleagues (38). More research is needed to determine the most effective approach of using sensory techniques to target multiple sensory systems as they address unique sensory processing patterns of a child that impact participation, as compared to applying a broad approach of general sensory intervention that is not targeted to unique sensory processing needs.

The use of *sensory techniques* targeting a single sensory system had limited evidence. Alternative seating did not have evidence to support its use if the goal was to improve functional behaviors or attention (32–34); another study demonstrated that fidget spinners had a negative effect on attention (43); and, finally, noise-cancelling headphones (44) were not found to impact auditory participation goals. However, Ikuta and colleagues (44) did find significant improvements in auditory-related goals with earmuff use. This indicates that particular tools, headphones vs. earmuffs, may change effectiveness and prompts the need for further research to examine if the tool or the person's preferences are more impactful when examining the effectiveness of SBIs.

An exception to the generally weak evidence for interventions targeting a single sensory system is the provision of deep-pressure tactile stimulation, which has shown more promising results. Deep-pressure tactile stimulation has modulating effects on the nervous system via decreasing sympathetic arousal and increasing parasympathetic activity (60, 61). Two studies found sleep behavior improvements, one with use of weighted blankets during sleep (25) and one with massage and joint compressions prior to bedtime (28). Nielsen and colleagues (27) noted improvements in areas such as learning, attention, and coping with the use of a vest for deep-pressure tactile stimulation, but results were not significant. All three of these studies focused on children with ADHD or SI/SP challenges, suggesting that deep-tactile pressure tactile stimulation may be a useful tool in supporting occupational outcomes with these populations. These findings are consistent with previous research findings supporting use of Qigong massage (14, 15), suggesting that deep-pressure tactile stimulation should be included as part of intervention recommendations when indicated, such as for improving sleep and self-regulation, for children with SI/SP challenges.

For the *caregiver-focused interventions* outcomes, this review builds on the emerging evidence found by Miller-Kuhanack and Watling (16) which supports group caregiver training on sensory processing and sensory motor strategies for children with ASD. Two studies in this current review evaluated *caregiver-focused interventions* for children with ADHD and SPD, providing additional evidence that group training in sensory–motor strategies can enhance caregiver awareness and their ability to implement these approaches effectively (50, 51). Overall, the result of this systematic review supports the use of caregiver training to implement sensory strategies across populations of children with SI/SP challenges. However, the outcome measures across these studies varied. While two studies investigated outcomes related to quality of life and participation (52, 53), two of the studies demonstrated improvements in caregiver knowledge when using sensory-based strategies at home (50, 51). Therefore, although caregiver training has strong evidence to support its use, the impact on the client and family requires continued research.

Finally, there remains a dearth of research investigating the effectiveness of *sensory environmental modifications*. There was only one study that met the criteria for this systematic review (56), which focused on a sensory-adapted dental environment, finding improved behavior scores as compared to the standard dental environment. There is not enough research examining how sensory environmental modifications specifically support client participation. Future investigation in this domain may need to focus on community-engaged research that explores how environmental adaptations can enhance meaningful engagement for individuals with SI/SP challenges. For example, Silverman and Tyszka (62) conducted a qualitative community-based action research study to investigate the benefits of sensory-friendly programming for museum participation in children and adults with SI/SP challenges. Findings indicated improvements in the quality and duration of museum visits for individuals with SI/SP challenges. Contextual models such as the Ecology of Human Performance (63) may be useful in guiding research that examines the impact of sensory modifications to environments for improved participation. Sensory-friendly spaces and sensory environmental adaptations continue to be popular in contemporary culture but lack research on effectiveness related to improving participation. Understanding how the physical and social environment influences participation of individuals, groups, and populations is a key consideration for future research on the effectiveness or the impact of sensory environmental modifications on participation.

One of the key challenges in researching SBIs is the absence of clear implementation guidelines and inconsistency in reported outcome measures across studies. SBIs aim to alter the level of physiological arousal to provide a better match between the person and the task, thereby improving behavior and self-regulation (11). Many outcome measures on the effectiveness of SBIs focused on observable changes in behavior. Consequently, the outcome measures included in these studies relied on observable changes in behavior rather than considering internal physiological changes that might support occupational performance. While behavioral regulation requires the cognitive functions of attention, working memory, and inhibition (64), SBIs primarily focus on the organizing impact of sensory input to support modulation (11, 65). Therefore, there may be a mismatch between the intent of the SBI and the outcome measures used to measure the impact. Future research may consider including measures of physiological arousal such as heart rate variability, skin conductance, respiration rates, etc. [e.g., (66, 67)] in combination with participation-based outcomes.

In addition, no study examined the child's perspective on the use of sensory techniques, which is a key outcome. Sensory techniques are designed for the purpose of altering physiological states of arousal to improve participation in given tasks (11). If a child reports (or has the perception) that an SBI, such as alternative seating, improves their ability to participate in the task, then it may be considered an effective intervention. Assessing goal attainment from the individual's perspective, rather than relying on behavioral outcomes, may be a better indicator of the effectiveness of the sensory technique. Future research with SBIs should focus on systematic measurement of effectiveness and consistent outcome measurement to strengthen the body of literature in this often-requested intervention. It is important that outcomes consider the perspective of the child who is receiving the intervention. Children often report feelings of being unheard, unsupported, and disrespected in their own care (68). Including outcomes that reflect the child's perspective, rather than relying solely on proxy reports or behavioral observations, allows practitioners to tailor care to a more child-centered approach.

Finally, it is not clear if all studies included in this review had an occupational therapist guiding the intervention. Occupational therapists are considered the experts in supporting children with SI/SP challenges. They have comprehensive training and provide a unique understanding of the theoretical framework that guides evaluation of sensory processing needs and intervention decisions.

## Limitations and future research

This systematic review was limited to studies of sensory-based interventions (SBIs) published in English between 2015 and January 2024. As with any review, some studies may have been missed or published after the search date. A primary limitation of the evidence is the lack of consistency in how, when, and under what conditions SBIs were implemented. While SBIs are based on sensory integration theory, there is no fidelity measure to guide practice, and most interventions lack manuals or standardized guidance. Future research should clarify how sensory strategies, tools, and equipment are utilized and establish replicable implementation parameters. This would allow practitioners to develop clearer guidelines for SBI use and purpose.

Another limitation is the absence of subjective or objective client perceptions. Outcomes were largely observable performance or proxy reports, despite children often reporting feeling unheard in healthcare (68). Future research should examine the impact of sensory environmental modifications on learning and engagement and expand studies of caregiver training to include educators and employers. Research is needed across environments and the lifespan.

## Conclusion

This review highlights strong evidence for caregiver training on SBIs and the use of deep pressure tactile input to support functional outcomes for children and youth with SI/SP challenges. Evidence for a sensory technique targeting only one sensory system is limited and mixed, but when SBIs are matched to a child's unique sensory processing needs, they can support meaningful participation. Minimal evidence exists for modifying sensory environments, emphasizing the need to consider individual preferences and continued research. A collaborative effort between occupational therapy and other professionals should aim to enhance research quality, establish clear implementation guidelines, and ensure the effective translation of findings into practice to support participation of individuals with SI/SP challenges across settings.

### Implications

The findings of this systematic review have the following implications:

- The evidence indicates that caregiver training or coaching should be considered as part of any sensory intervention.
- SBIs may be more effective when implemented as part of a larger intervention plan, rather than as a standalone intervention.
- SBIs interventions should be matched to the unique sensory needs of an individual. Therefore, a clear assessment of sensory processing as part of a comprehensive evaluation to identify the client's needs should be performed by a trained practitioner to guide the intervention process.
- Sensory environmental modifications need more research to determine their effectiveness for individuals, groups, and populations.
- Continued research is needed to clarify the benefits of SBIs and standardized procedures should be developed so that SBI implementations are replicable and evidence-based.

## Data Availability

The original contributions presented in the study are included in the article/Supplementary Material, further inquiries can be directed to the corresponding author.
