# Supplementary material for: Systematic review of sensory-based interventions for children and youth (2015–2024)
Source: Front Pediatr. 2025 Nov 13;13:1720179. doi: 10.3389/fped.2025.1720179 (PMC12658592; doi:10.3389/fped.2025.1720179)
Supplement: Supplementary file 1 [file Datasheet1.docx]

*Evidence Table*

| **Author/Year** | **Level of Evidence**  **Study Design**  **Risk of Bias** | **Participants**  **Inclusion Criteria**  **Study Setting** | **Intervention and Control Groups** | **Outcome Measures** | **Results** |
| --- | --- | --- | --- | --- | --- |
| Sensory Techniques: Alternative Seating | | | | | |
| Benson et al., 2019  https://doi.org/10.1080/19411243.2019.1672606 | Level II  SSD: ABAB  *Risk of Bias*  Moderate | *Participants*  *N* = 4*;* 4.2-4.6 yr with ASD; 3 boys, 1 girl  *Inclusion Criteria*  Preschool children attending the LEAP program  *Intervention Setting*  Preschool  *Country*  United States | *Intervention*  Use of alternative seating (T-stool or cube chair)  A: Wks 1 & 3, no seat  B: Wks 2 & 4, alternative seat  *Control Group*  n/a (SSR) | *Attention and In-seat Behavior*: Video recording of morning circle over 4 wks, 2xs/wk for 10 min ea; observation of duration based on operational definitions of attention (e.g., answering, asking, pointing, raising hand, concentration on some feature of the process) and in-seat behaviors (e.g., contact with the seat with some part of body, breaking seat contact to engage with instructional task, returning to seat with one cue); visual analysis using 2 standard deviation band method | *Significant Findings*  None  *Nonsignificant Findings*  Duration of attention and in seat behaviors improved, but results were not significant for any participant |
| Krombach & Miltenberger, 2020  https://doi.org/10.1007/s10803-019-04283-8 | Level II  SSD: ABC  *Risk of Bias*  Moderate | *Participants*  *N* = 4; 4-12 yr, with ASD  and intellectual disability that had difficulties remaining seated more than 5 min  *Inclusion Criteria*  Students with ASD, intellectual disability, difficulty in attending during seated tasks, ability to sit upright on a ball without assistance, receptive language, and capable of complying with one-step directions  *Intervention Setting*  Home  *Country*  United States | *Intervention*  Sitting on a stability ball  A - Baseline  B - Intervention  15-20 wks; 2xs/wk intervention session (sessions ranged from 5-16 minutes in length, dependent on child)  C - Choice  *Control Group*  n/a (SSR) | *In-seat Behavior and Attention:* Videotaped observation of duration of the 2 behaviors based on operational definitions of in-seat behavior (e.g., buttocks in contact with the ball, ball in contact with the floor, one foot on the floor) and attention (e.g., appropriate interaction with materials per directive; responding to or looking at speaker, not oriented to other activities or items). | *Significant Findings*  None  *Nonsignificant Findings*  Attention and in-seat behavior percentage of time improved during intervention phase (ball chair) for all 4 children |
| Macphee et al., 2019  https://doi.org/10.17105/SPR-2017-0151.V48-3 | Level II  2x3 Within Subjects Design  *Risk of Bias*  Moderate | *Participants*  *N* = 64; ages 5-12 yr (*M* = 8.14 years; (82.8% male), with ADHD  *Inclusion Criteria*  Children with diagnoses of ADHD, IQ ≥80, without ASD, no current treatment with psychotropic medications, and no noted intolerability of psychostimulant medication.  *Intervention Setting*  School  *Country*  United States | *Intervention*  Sitting on stability ball for duration of class; wearing weighted vest for duration of class; 6 wks; 1 hr/wk during Summer Treatment Program;  received medication or placebo  *Control Group*  n/a (SSR) | *Classroom Rule Following and Accuracy of Seatwork Productivity*  Teacher recorded frequencies of classroom rule (e.g., be respectful, raise hand, remain in seat, work quietly) violations and the number of worksheet items a child could correctly complete in a 30 min period | *Significant Findings*  Statistically significant differences in the medication group for increased frequency of following rules (p = 0.032) and improved seatwork productivity (p < 0.001)  *Nonsignificant Findings*  No statistically significant improvements in the frequency of following classroom rules or seatwork productivity with either stability ball or weighted vest |
| Sensory Techniques: Multiple Sensory Techniques | | | | | |
| Benson et al., 2020  https://doi.org/10.1080/19411243.2020.1732262 | Level II  SSD: BABA  *Risk of Bias*  Moderate | *Participants*  *N* = 3; 4 yr, 3 mos - 5 yr, 2 mos with ASD; all males  *Inclusion Criteria*  Children with ASD in a convenience sample integrated preschool classroom    *Intervention Setting*  Preschool  *Country*  United States | *Intervention*  B: Sensorimotor phase (sensorimotor activities designed by OT for ea student’s sensory needs)  A: Non-sensorimotor phase  4 weeks (2 intervention phases and 2 non-intervention phases);  B: 15 min ea preschool circle time  A: no intervention  *Control Group*  n/a (SSR) | *Attention and In-seat Behavior: V*ideotape of morning circle over 4 wks, 2xs/wk for the first 10 min after the child was seated; observation of duration based on operational definitions of attention (e.g., answering or asking questions, participation upon request, raising hand, no more than 2 teacher cues to complete instructional directive) and in-seat behavior (e.g., maintaining physical contact with seating option, breaking contact to engage in instructional task if appropriate, returning to seat with one teacher cue). | *Significant Findings*  Only 1 of the 3 participants had significant effect for in-seat behavior and attention improvement in both sensorimotor phases (100% PND) with p=0.05 set as significant  *Nonsignificant Findings*  Although improvements were observed, statistically significant improvements in attention or in-seat behavior were not observed in 2 of 3 participants. |
| Benson et al., 2022  https://doi.org/10.1080/19411243.2021.2003734 | Level II  SSD: ABAB  *Risk of Bias*  Moderate | *Participants*  *N* = 7; ages 8-12 yr, with ASD; 5 males, 2 females  *Inclusion Criteria*  Convenience sample from private, special education school; ASD diagnosis and identified as having sensory processing challenges that impacted task performance  *Intervention Setting*  School  *Country*  United States | *Intervention*  2 consecutive weeks;  A: Baseline (days 1 & 2)  B: Intervention (days 4 & 5); Sensory Activity Schedule (SAS) embedded in workstation schedule during independent work time as part of TEAACH teaching model, inclusive of vestibular, proprioceptive, oral motor, and visual  *Control Group*  n/a (SSR) | *On-task Behavior*  6 observations ea day of phases; 15 min per observation; duration of on task behavior during independent work time per operational definition (e.g., interaction with materials for targeted behaviors, task completion) and the number of cues students needed to engage in on-task behavior | *Significant Findings*  Significant decrease in number cues for 4 of 7 participants (p=0.05)  Significant difference in mean number of cues provided for all participants from phase A1 to B1 (p=0.01)  *Nonsignificant Findings*  Non-effective on PND for all participants (PND 70% or higher considered effective) |
| Pingale et al., 2022  https://doi.org/10.1080/19411243.2021.1941492 | Level II  SSD: ABCA  *Risk of Bias*  Moderate | *Participants*  *N* = 5 (5.2 - 10 yr; all male);  *Inclusion Criteria*  Children ages 4-11 yr in pre-k through 4th grade; documented SI/SP challenges*;* no physical  limitations or disabilities, not taking stimulants, anti-anxiety, or anticonvulsant medications, and not having received sensory diets within 6 mo    *Intervention Setting*  School  *Country*  United States | *Intervention*  28 days (7 days for ea phase)  A: regular school day  B: age-appropriate fine-motor and visual activities that are typically used in classrooms per the schedule  C: Sensory diet (SD) including customized; tactile, vestibular, and proprioceptive activities; 3xs/day for 5-7 min  *Control Group*  n/a (SSD design) | *Individualized Target Behaviors*  Video recorded for  15 min interval during a group activity; frequency counts/duration of individualized target behaviors addressing sensory processing, psychosocial skills, and classroom engagement goals; each participant had individualized definitions for the three categories of targeted behaviors | *Significant Findings*  P1: significant for phases B-C for classroom engagement (p = .01) and C-A2 for psychosocial skills and classroom engagement (p = .01)  P2: significant for phase B-C for psychosocial skills and classroom engagement and C-A2 for sensory processing skills (p = .01)  P3: significant for phase B-C for sensory processing skills, psychosocial skills, and classroom engagement and C-A2 for sensory processing skills (p = .01)  P4: significant for phase B-C for psychosocial skills and in C-A2 for psychosocial skills and classroom engagement  (p = .01)  P5: significant for phase B-C for sensory processing skills, psychosocial skills, and classroom engagement and C-A2 for psychosocial skills and classroom engagement (p = .01)  *Nonsignificant Findings*  All participants showed a decrease in problematic behaviors for all categories with introduction of SD phase and increase upon withdrawal  Additional results:  P2: psychosocial skills and classroom engagement improved approaching significance for phase C-A2; contra-therapeutic responses for classroom engagement upon withdrawal of SD phase  P3: classroom engagement improved approaching significance for phase B-C |
| Voola & Kumair, 2022  https://doi.org/10.35841/0971-9032.26.4.1381-1385 | Level II  Quasi-experimental pretest/posttest  *Risk of Bias*  Moderate | *Participants*  *N* = 10 (9 male, 1 female); 6-12 yr, with ADHD  *Inclusion Criteria*  Children 6-12 yr with diagnosed ADHD and SI/SP    *Intervention Setting*  Clinic and sensory garden  *Country*  India | *Intervention*  *N* = 5  Nature-based sensory garden (SG); 12 wks, 3xs/wk for a total of 46 sessions (45 min ea session);  plus indoor sensory integration (SI)  *Control Group*  *N* = 5  only received indoor SI therapy | *Functional Skills*  *Weiss Functional Impairment Rating Scale* (WFIRS) | *Significant Findings*  Improvements in the SG intervention group on WFIRS (p < 0.01) for functional behavior  *Nonsignificant Findings*  None |
| Koshy et al., 2018  https://doi.org/10.4103/0445-7706.244548 | Level II  Quasi-experimental  *Risk of Bias*  High | *Participants*  *N* = 21; 3-10 yr with sensory processing disorder and toileting difficulties  *Inclusion Criteria*  Children 3-10 yr with complaints of toileting problems and conditions such as autism, ADHD, learning disabilities, fragile X, and emotional problems but without physical dysfunction, visual, or hearing impairments; must not be receiving other laxative therapy, enemas, and  suppositories, biofeedback, or medication for constipation.  *Intervention Setting*  Clinic and Home  *Country*  India | *Intervention*  *N* = 10  SI treatment and behavioral modification therapy; 4 mos, 2xs/wk (45 min/session; remaining days at home by parents  *Control*  *N* = 11  Behavior modification therapy | *Toileting*  *Canadian Occupational Performance Measure* (COPM) | *Significant Findings*  Significant difference (significance set at p<.05) for experimental group (p=0.005)  and control group (p=0.004) for performance and satisfaction on COPM toileting tasks  Medium effect size for experimental group; small effect size for control  *Nonsignificant Findings*  None |
| Sensory Techniques: Sensory Tools | | | | | |
| Durgut et al., 2020  https://doi.org/10.3233/NRE-203040 | Level 1  RCT  *Risk of Bias*  Low | *Participants*  *N* = 30, 15 per group (3 girls, 12 boys); randomly assigned to Treadmill Training (TT) (*M* age = 8.33 yr) or TT + Whole Body Vibrational Training (WBVT) (*M* age = 7.93 yr)  *Inclusion Criteria*  Children ages 7-11 diagnosed with ADHD with no previous treatment for ADHD or comorbid conditions; children and parents with reading skills  *Intervention Setting*  Outpatient clinic  *Country*  Turkey | *Intervention*  TT + WBVT; 3xs/wk for 8 wks; TT was 45 min and WBVT in a standing position for 15 min w/o hand  support on a sinusoidal vibration platform  *Control Group*  3xs/wk for 8 wks; TT only (45 min) | *Quality of Life* Pediatric Quality of Life Inventory (PedsQL)  *Executive Functioning* Behavior Rating Inventory of Executive Function (BRIEF)  *Attention*  Conner’s Rating Scale  Stroop test TBAG form (STP-TBAG) | *Significant Findings*  Statistically significant improvements (p<0.05) for both groups on PedsQL both children and parent forms, BRIEF (executive functioning), Conner’s Rating scale (attention), and STP-TBAG form for intervention group (attention)  *Nonsignificant Findings*  None |
| Graziano et al., 2020  https://doi.org/10.1177/1087054718770009 | Level II  SSD: ABAB  *Risk of Bias*  Moderate | *Participants*  *N* = 60 (*M* age = 4.86 yr), 75% male with ADHD;  Data collected for 4 random children in each classroom, rotated across 3 days for a total of 48 children  *Inclusion Criteria*  Diagnosis of ADHD, enrollment in school in the prior yr, IQ of ≥ 70, no diagnosis of ASD or psychotic disorder, ability to attend 8-week program  *Intervention Setting*  School  *Country*  United States | *Intervention*  Use of fidget spinners during the school day of a Summer Treatment Program (STP)  A: Baseline (wks 1 and 6 of STP)  B: Fidget spinner (wks 2 and 7 of STP); regular behavior interventions  *Control Group*  n/a (SSR) | *Gross Motor Movements*  Accelerometers worn during both phases  *Behavior and Attention in the Classroom:* Videotape for 5 min during ELA classroom to attain frequency counts rule “violations” as part of the school’s standard rule structure | *Significant Findings*  First intervention phase:  Significantly greater number of attention violations with use of fidget spinner (p<0.01)  *Nonsignificant Findings*  Gross motor movements not statistically significant in either phase (p=0.07)  No effect on second phase for behavior in the classroom significance at p<0.05) |
| Ikuta et al., 2016  https://doi.org/10.1016/j.hkjot.2016.09.001 | Level II  Within subjects crossover experimental design  *Risk of Bias*  Low | *Participants*  *N* = 21; 4-16 yr; 16 boys and 5 girls with ASD  *Inclusion Criteria*  Children diagnosed with autistic disorder, Asperger’s disorder, or pervasive developmental disorder; 3-17 yr; hyper-reactivity to auditory stimuli  *Intervention Setting*  Home and school  *Country*  Japan | *Intervention*  Wearing earmuffs and noise canceling (NC) headphones worn part of the day.  A - Baseline  B - Earmuff  C - NC headphones  4 wks (2 wks earmuff use and 2 wks NC headphone use); random order for various amounts of time  *Control Group*  n/a (SSR) | *Behaviors Related to Auditory Stimuli*  Goal Attainment Scaling (GAS) | *Significant Findings*  Improved GAS (participation behaviors) related to auditory stimuli for earmuff period as compared to the baseline/control period (p = 0.006)  *Nonsignificant Findings*  No statistically significant participation behavior changes in NC headphone phase compared to the baseline phase, or between earmuff and NC headphone phases |
| Sensory Techniques: Deep Pressure Tactile | | | | | |
| Lönn et al., 2024  https://doi.org/10.1111/jsr.13990 | Level I  RCT  crossover design  *Risk of Bias*  Low | *Participants*  *N* = 91; 6-14 yr (M = 9 yr); 57% boys; ADHD  *Inclusion Criteria*  Children with ADHD without significant comorbidities; sleep problems, stable medication, lack of or discontinuation of melatonin medication, and no prior use of WBs.  *Intervention Setting*  Home  *Country*  Sweden | *Intervention*  Weighted blanket (WB) for sleep;  8 wks; 4 + 4 wks; randomized to start with weighted blanket (WB) or control blanket (CB)  *Control Group*  CB (non-weighted; 2kg) for sleep | *Sleep Onset Latency, Total Sleep Time, Sleep Efficiency, and Wake After Sleep Onset*  Actigraph  Sleep questionnaire | *Significant Findings*  Intervention group statistically significant improvements in total sleep time (p = 0.027); sleep efficiency (p = 0.038); and wake after sleep onset (p = 0.015)  (Significance set at p<0.05)  *Nonsignificant Findings*  No effect on sleep onset latency |
| Lu et al., 2019  https://doi.org/10.1080/17518423.2018.1537317 | Level I  RCT  *Risk of Bias*  Moderate | *Participants*  *N* = 36 children; ages 1-3 years, with developmental delay; stratified by age (7–12 mos vs. 13–36 mos) and motor development quotient (DQ) on the CDIIT-DT (DQ ≥ 70 vs. DQ < 70)  *Inclusion Criteria* Children between the ages of 7 and 36 mos, diagnosis of DD, one or more delays on the *Comprehensive Development Inventory for Infants and Toddlers Diagnostic Test* (CDITT-DT), body weight below 50th percentile for age, and native Chinese speaking primary caregiver.  *Intervention Setting*  Clinic or Home  *Country*  Taiwan | *Intervention*  *N* = 18 (*M* age = (24.1; 55.6% boys);  Face and body massage by author (OT and certified infant massage instructor) as tolerated; 2xs/wk for 12 wks; 20 min ea session; with routine rehabilitation intervention  *Control*  *N* = 18 (*M* age = 23.4; 72% boys);  Routine rehabilitation intervention | *Motor, Language, Self-help, and Social Skills*  CDIIT-DT  *Sleep*  Sleep questionnaire | *Significant Findings*  Intervention group statistically significant improvement in total motor score (p=0.023)  Significance set at p=0.05  *Nonsignificant Findings*  No significant effect was found in the scores on CDITT-DT subtests for fine motor, cognitive, language, self-help, or social; gross motor approached significance (p=0.047); No significant difference in sleep patterns for intervention or control |
| Nielsen et al., 2023  https://doi.org/10.3390/children10111800 | Level II  Quasi-experimental pretest/posttest  *Risk of Bias*  Low | *Participants*  *N* = 70; 6-12 yr (*M* age = 8.93; 51 boys, 19 girls), exhibiting sensory seeking behaviors  *Inclusion Criteria*  Documented SI/SP challenges with no other significant diagnosis  *Intervention Setting*  School  *Country*  Finland | *Intervention*  3 wks of wearing the vest for the first 90 min at the start of every school day; students then invited to use the vest as much as they liked, both at home and at school  Teachers received 90-min lecture about dysfunctions in sensory processing (DSP), the use of Protac My Fit ball vest, and how to support childrens’ attention in the classroom.  *Control Group*  None | *Attention, Body Perception, Coping Skills, and Learning*  Five to Fifteen (FTF) parental survey; Strengths and Difficulties Questionnaire  (SDQ) teacher survey | *Significant Findings*  No statistically significant results (significance set at p < 0.05)  *Nonsignificant Findings*  Reported increases in attention, improvements in body perception, and improvements in coping skills in learning affecting everyday life but not statistically significant |
| Spira, 2021  https://doi.org/10.1108/IJOT-09-2020-0014 | Level II  Quasi-experimental  pretest/posttest with non-blinded randomization  *Risk of Bias*  Low | *Participants*  *N* = 50; 5.2 to 10.8 yr with sensory processing differences and sleep difficulties  *Inclusion Criteria*  Children ages 5.0-11.11 yr with significant SI/SP difficulties concurrent significant behavioral sleep difficulties no comorbid diagnoses of ASD, intellectual disabilities, or psychiatric disorders  *Intervention Setting*  Home  *Country*  Israel | *Intervention*  *N* = 25 (16  males, 9 females); massage with joint compressions; 4 wks for 15-20 min nightly  *Control Group*  *N* = 25 (15 males, 10 females); bedtime story | *Sleep*  Goal Attainment Scaling (GAS)’  Child Sleep Habits Questionnaire (CSHQ) | *Significant Findings*  Statistically significant improvements in sleep behaviors (p < 0.001); parasomnias (p < 0.01); and sleep duration (p < 0.05)  Decreases in sleep anxiety (p < 0.05); daytime sleepiness (p < 0.05); and GAS (p < 0.0001)  *Nonsignificant Findings*  Sleep disordered breathing did not show significant improvement |
| Caregiver Training | | | | | |
| Heyburn et al., 2023  https://doi.org/10.1080/01942638.2022.2104151 | Level II  Quasi-experimental pretest/posttest  *Risk of Bias*  Moderate | *Participants*  *N* = 95; Parents of children ages 2-18 yr (*M* age = 6 yr; 72% male) with sensory concerns  *Inclusion Criteria*  Parents of children referred for children’s allied health program for sensory concerns, registered for intervention  *Intervention Setting*  Therapy center  *Country*  Canada | *Intervention*  Sensory in-service;  structured, 2 hr, group-based (up to 15 families), caregiver education intervention,  *Control Group*  No control | *Occupational Performance*  *Canadian Occupational Performance Measure* (COPM)  *Caregiver Knowledge of Sensory Processing and How to Support Their Child*  *Sensory Processing and Your Child* Caregiver Knowledge Questionnaire | *Significant Findings*  COPM improvements (individualized occupational performance goals) (p<0.001); COPM Satisfaction (p<0.001) and Caregiver knowledge (p<0.001).  Large effect size for all (EF=.94-1.00)  *Nonsignificant Findings*  None |
| Padmanabha et al. 2019  https://doi.org/10.1007/s12098-018-2747-4 | Level I  RCT  *Risk of Bias*  Moderate | *Participants*  *N* = 40; children 3-12 yr, with ASD and sensory processing differences  *Inclusion Criteria*  Age 3-12 yr; confirmed sensory processing difficulties; no active seizures; no tuberous sclerosis  *Intervention Setting*  Home and clinic  *Country*  India | *Intervention*  *N* = 21(*M* age = 48 mos; 76% male);  12 wks; 5 days/wk (45-60 min sessions) of sensory intervention group therapy, plus home-based sensory interventions by caregiver  *Control Group*  *N* = 19 (*M* age = 48 mos; 94% male);  standard therapy (speech therapy and ABA therapy) | *Quality of Life*  Pediatric Quality of Life Inventory (PedsQL)  *Behavior and Emotional Function*  Children’s Global Assessment Scale (CGAS) | *Significant Findings*  PedsQL: improvement in quality of life for intervention group (p<0.008)  CGAS: improvement (behavioral and emotional functioning) (p < 0.011)  *Nonsignificant Findings*  None |
| Pashazadeh Azari et al., 2019  https://doi.org/10.22037/ijcn.v13i4.21156 | Level I  RCT with a mixed within-between-  subjects design  *Risk of Bias*  Moderate | *Participants*  *N* = 38 (31 completed all phases); parents of children ages 3-10 yr, with ASD diagnosis and sensory processing differences  *Inclusion Criteria*  Parents of children 3-10 yr at point of recruitment; prior ASD diagnosis; at least one sensory pattern out of typical range on *Short Sensory Profile - 2*  *Intervention Setting*  Clinic  *Country*  Iran | *Intervention*  *N* = 19 (parents of children *M* age = 6.5 yrs); 16 completed all phases of study;  Contextual Intervention adapted for ASD (CI-ASD) with three components: 1) sensory processing knowledge, 2) coaching, 3) social support  11 wks; 2 group training sessions in CI-ASD and 10 weekly, individual coaching sessions (45 min ea)  *Control Group*  *N* = 19; (parents of children *M* age = 7.12 yrs) 15 completed all phases of study;  Wait list; usual treatment; all participants received other services (ABA, speech therapy, group therapy, medication) throughout the study | *Occupational Performance*  Canadian Occupational Performance Measure (COPM)  *Goal Attainment in functional goals and routines*  Goal attainment scaling (GAS)  *Parent Efficacy*  Parenting Sense of Efficacy Measure (PSEM) | *Significant Findings*  COPM improvements (individualized goals) for performance (post p = 0.011; follow-up p < 0.001); and Caregiver satisfaction (post p = 0.004; follow-up p < 0.001)  GAS improvements for individualized goals (post p < 0.001; follow-up p < 0.001)  PSEM improvements in parent efficacy (post p = 0.011; follow-up p = 0.043)  *Nonsignificant Findings*  None |
| Mah & Doherty, 2021  https://doi.org/10.1007/s10826-021-01900-y | Level II  Pretest/posttest with randomization  *Risk of Bias*  Low | *Participants*  *N* =16; 8-12 yr (*M* age = 9.37; (93.8% male), with ADHD and SPD, and their guardian  *Inclusion Criteria*  Able to participate in a group setting; speak and understand English; be  stable on medication for ADHD if taking any; not have unmanaged ADHD symptoms, psychosis, an intellectual disability, or autism spectrum disorder; have not completed previous “How Does Your Engine Run? - The Alert Program® for Self Regulation (AP) groups  *Intervention Setting*  Clinic  *Country*  Canada | *Intervention*  AP; 4 consecutive wks; one 2 hr session/wk; facilitated by OT (who received 2-day in class training on AP) and nurse clinician (trained by OT); N=8 families  *Control Group*  Wait list  N=8 | *Knowledge of Sensorimotor*  Sensory and Motor Strategies Questionnaire (SMSQ)  *Occupational Performance*  *Canadian Occupational Performance Measure* (COPM) | *Significant Findings*  Significant increase in knowledge of sensorimotor strategies on SMSQ (p < 0.001)  Large effect in knowledge and sensorimotor strategies (>0.14)  *Nonsignificant Findings*  No significance on COPM |
| Mah et al., 2023  https://doi.org/10.1177/13591045231162680 | Level II  Quasi-experimental pretest/posttest  *Risk of Bias*  Moderate | *Participants*  *N* = 27; children 8-12 yr (*M* age = 9.96; 22 male, 5 female), with ADHD and SPD, and their guardians  *Inclusion Criteria*  Diagnosis of ADHD; able to participate in a group setting; stable on ADHD meds if taking any; no unmanaged ADHD  symptoms, psychosis, intellectual disability, or autism spectrum disorder; speak and understand English; had not previously completed an AP group; no previous or current behavioral parent interventions  *Intervention Setting*  Clinic  *Country*  Canada | *Intervention*  AP; 8 consecutive wks; one 90 min session/wk; primarily facilitated by OT (who received 2-day in class training on AP) and supported by nurse clinician or psychologist; parent-only psychoeducation sessions occurred at pre- and post-AP (2 sessions)  *Control Group*  None | *Knowledge of Sensorimotor*  *Sensory and Motor Strategies Questionnaire* (SMSQ)  *Visual Attention*  Frequency counts of observed problem behaviors (i.e., off-task behaviors, fidgeting, vocalizing, plays with objects, out of seat) during videotaped visual related tasks | *Significant Findings*  Statistically significant increase in knowledge and use of sensorimotor strategies for parents at home (p < 0.001), but not for teachers.  Large effect size (> 0.14)  Child problem behaviors observed on the visual attention task significantly increased from pre-AP to post-AP (p < 0.001)  *Nonsignificant Findings*  None |
| Sensory Environmental Modifications | | | | | |
| Kim et al., 2019  https://doi.org/10.1111/scd.12360 | Level II  Crossover study  *Risk of Bias*  Moderate | *Participants*  *N*= 22; children 6-21 yr (62% male), 57% non-verbal, 71% probable sensory modulation disorder  *Inclusion Criteria*  Sample of convenience from university clinic for oral health; English speaking patient or guardian; diagnosed with a developmental delay and sensory modulation disorder    *Intervention Setting*  Dental office  *Country*  United States | *Intervention*  Crossover study  Sensory adapted dental environment (SADE) 36 visits; randomly selected for one environment, followed by opposite environment 3 mos later  *Control Group*  Regular dental environment (RDE) | *Behaviors in Dental Office*  *Frankl Scale*  *Physiological Signs*  Heart rate and oxygen saturation | *Significant Findings*  Frankl behavior (indicating attitude towards dental treatment) score for SADE was significantly higher compared to Frankl score for RDE (p = 0.0368)  *Nonsignificant Findings*  Paired analysis of Frankl behavior rating scale approached significance (p=0.07);  No significant difference in physiological (heartrate and oxygen saturation) outcomes |
